# Supplementary material for: A computational approach to evaluate how molecular mechanisms impact large-scale brain activity
Source: Nat Comput Sci. 2025 May 28;5(5):405–17. doi: 10.1038/s43588-025-00796-8 (PMC12119344; doi:10.1038/s43588-025-00796-8)
Supplement: Supplementary file 1 — Supplementary Tables 1–7 and details on statistical analysis. [file 43588_2025_796_MOESM1_ESM.pdf]

nature computational science

Supplementary information

https://doi.org/10.1038/s43588-025-00796-8

A computational approach to evaluate how molecular mechanisms impact large-scale brain activity

In the format provided by the authors and unedited

Supplementary Information

1 Fitting parameters

Cell Type	P_0	$P_{\mu V}$	$P_{\sigma V}$	$P^V_{\tau N}$	$P^2_{\mu V}$	$P^2_{\sigma V}$	$P^V_{(\tau N})^2$	$P_{\mu V}\sigma V$	$P_{\mu V}\tau N^V$	$P_{\sigma V}\tau N$
RS-cell	-50.17	4.52	-7.94	-2.08	-0.55	3.42	-11.56	1.95	2.74	-10.67
FS-cell	-51.85	6.16	-14.04	1.67	-2.06	3.18	-31.13	6.57	1.72	-45.16

Supplementary Table 1: Fitting parameters expressed in mV

2 Model parameters

Supplementary Table 2: Model Parameters

Parameter name	Symbol	Awake	NMDA-block	$\mathbf{GABA}_A$ agonism	NREM sleep	Unit
**Cellular Properties**						
Leak conductance	g_L		10			nS
Membrane capacitance	C_m		200			pF
Resting voltage	v_{rest}		-65			mV
Action Potential threshold	v_{thr}		-50			mV
Refractory period	T_{refr}		5			ms
Adaptation time constant	τ_w		500			ms
Excitatory Neuron						
Leak reversal potential	E_L		-64			mV
Spike sharpness	Δ_e		2			mV
Adaptation current increment	b_e	5	30	30	120	pA
Adaptation conductance	a_e		0			nS
Inhibitory Neuron						
Leak reversal potential	E_L		-65			mV
Spike sharpness	Δ_i		0.5			mV
Adaptation current increment	b_i		0			pA
Adaptation conductance	a_i		0			ns
**Synaptic Properties**						
Excitatory Neuron						
Reversal potential	E_e		0			mV
Quantal conductance	Q_e		1.5			nS
Decay time of synaptic conductance	τ_e	5	3.75	5	5	ms
Inhibitory Neuron						
Reversal potential	E_i		-80			mV
Quantal conductance	Q_i		5			nS
Decay time of synaptic conductance	τ_i	5	5	7	5	ms
**Network Properties**						
Total network size	N		10000			
Global Coupling Factor	G		0.3			
Signal Propagation Speed	V_c		4.0			mm/ms
Connectivity probability	p		0.05			
Fraction of inhibitory cells	g_{ei}		0.2			

3 Statistical Analysis

3.1 Statistical analysis reported in Figure 5

The correlations between structural and functional connectivity correspond to the Pearson correlation between the vectorized structural and functional matrices.

Supplementary Table 3: Statistical Summary and Mann-Whitney U Test Results for the experimental data

**Statistic**	**Awake**	**Anesthesia**
Mean	0.2974	0.3304
Variance	0.0003	0.0007
Standard Deviation	0.0184	0.0272
Minimum	0.2582	0.2692
Maximum	0.3445	0.3835
Median	0.2956	0.3314
Q1 (25th percentile)	0.2882	0.3082
Q3 (75th percentile)	0.3049	0.3470
**Mann-Whitney U Test**	U statistic = 108.0, p-value = 2.6677e-05	

Supplementary Table 4: Statistical Summary and Mann-Whitney U Test Result for the simulated data

**Statistic**	**Awake**	**Anesthesia**
Mean	0.2998	0.3938
Variance	0.0002	0.0001
Standard Deviation	0.0141	0.0101
Minimum	0.2747	0.3771
Maximum	0.3184	0.4114
Median	0.2990	0.3923
Q1 (25th percentile)	0.2883	0.3874
Q3 (75th percentile)	0.3146	0.4013
**Mann-Whitney U Test**	U statistic = 256.0, p-value = 1.5449e-06	

3.2 Statistical analysis reported in Figure 6

Following the rejection of the Kruskal-Wallis ($H = 114.9, p = 9.5e-25$), we used the Conover post-hoc test to perform pairwise comparisons between the simulated different conditions (median and Interquartile range (IQR) for GABA$_A$: $0.109(0.149 - 0.076)$, NMDA-block: $0.099(0.135 - 0.078)$, NREM sleep: $0.152(0.198 - 0.086)$, Wakefulness: $0.360(0.378 - 0.345)$).

In Table 5, we report the p-values for each of the pairwise comparisons, adjusted for multiple comparisons using the Holm-Bonferroni method:

In Table 6, we also report the T-statistic of the Mann-Whitney U test between each pair of groups calculated as:

$$T = \frac{U - \frac{n_1 \cdot n_2}{2}}{\sqrt{\frac{n_1 \cdot n_2 \cdot (n_1 + n_2 + 1)}{12}}}$$

Supplementary Table 5: Pairwise p-values for comparisons of PCI values among conditions

	$GABA_A$-agonism	NMDA-block	NREM sleep	Wakefulness
$GABA_A$-agonism	1.0	5.83e-01	4.81e-03	1.06e-28
NMDA-block	5.83e-01	1.0	1.75e-02	5.73e-27
NREM sleep	4.81e-03	1.75e-02	1.0	1.2e-18
Wakefulness	1.06e-28	5.73e-27	1.2e-18	1.0

where U is the Mann-Whitney U statistic, and n_1 and n_2 are the sample sizes of the two groups compared.

Supplementary Table 6: Pairwise T-statistics for comparisons of PCI values among conditions

	$GABA_A$-agonism	NMDA-block	NREM sleep	Wakefulness
$GABA_A$-agonism	1.00	-0.05	-2.89	9.27
NMDA-block	-0.05	1.00	-2.36	8.10
NREM sleep	-2.89	-2.36	1.00	8.12
Wakefulness	9.27	8.10	8.12	1.00

Effect sizes were calculated using the T-statistics from the previous section as:

$$r = \frac{T}{\sqrt{2 \cdot n}}$$

where n is the sample size for each group. For each pairwise comparison, the effect size was computed and is summarized in Table 7:

Supplementary Table 7: Pairwise Effect Sizes (r) for comparisons of PCI values among conditions

	$GABA_A$-agonism	NMDA-block	NREM sleep	Wakefulness
$GABA_A$-agonism	1.00	-0.00	-0.26	0.85
NMDA-block	-0.00	1.00	-0.22	0.74
NREM sleep	-0.26	-0.22	1.00	0.74
Wakefulness	0.85	0.74	0.74	1.00
