# Supplementary material for: A computational approach to evaluate how molecular mechanisms impact large-scale brain activity
Source: Nat Comput Sci. 2025 May 28;5(5):405–17. doi: 10.1038/s43588-025-00796-8 (PMC12119344; doi:10.1038/s43588-025-00796-8)
Supplement: Supplementary file 2 — Reporting Summary [file 43588_2025_796_MOESM2_ESM.pdf]

nature portfolio

Corresponding author(s): Alain Destexhe

Last updated by author(s): Mar 12, 2025

Reporting Summary

Nature Portfolio wishes to improve the reproducibility of the work that we publish. This form provides structure for consistency and transparency in reporting. For further information on Nature Portfolio policies, see our Editorial Policies and the Editorial Policy Checklist.

Statistics

For all statistical analyses, confirm that the following items are present in the figure legend, table legend, main text, or Methods section.

n/a	Confirmed	
☐	☒	The exact sample size (*n*) for each experimental group/condition, given as a discrete number and unit of measurement
☒	☐	A statement on whether measurements were taken from distinct samples or whether the same sample was measured repeatedly
☐	☒	The statistical test(s) used AND whether they are one- or two-sided<br>*Only common tests should be described solely by name; describe more complex techniques in the Methods section.*
☒	☐	A description of all covariates tested
☐	☒	A description of any assumptions or corrections, such as tests of normality and adjustment for multiple comparisons
☐	☒	A full description of the statistical parameters including central tendency (e.g. means) or other basic estimates (e.g. regression coefficient) AND variation (e.g. standard deviation) or associated estimates of uncertainty (e.g. confidence intervals)
☐	☒	For null hypothesis testing, the test statistic (e.g. *F*, *t*, *r*) with confidence intervals, effect sizes, degrees of freedom and *P* value noted<br>*Give P values as exact values whenever suitable.*
☒	☐	For Bayesian analysis, information on the choice of priors and Markov chain Monte Carlo settings
☒	☐	For hierarchical and complex designs, identification of the appropriate level for tests and full reporting of outcomes
☐	☒	Estimates of effect sizes (e.g. Cohen's *d*, Pearson's *r*), indicating how they were calculated

Our web collection on statistics for biologists contains articles on many of the points above.

Software and code

Policy information about availability of computer code

Data collection	The code used for the simulations of this study was developed by our group. The code source of all simulations shown in this article is available online in https://github.com/mariasacha/paper_pipeline_hub/ where the exact versions of the packages used can be found in the requirements.txt. Our code use the Virtual Brain https://www.thevirtualbrain.org/tvb/zwei/home (version 2.9)
Data analysis	The code used for the simulations of this study was developed by our group. The code source of all simulations shown in this article is available online in https://github.com/mariasacha/paper_pipeline_hub/ where the exact versions of the packages used can be found in the requirements.txt. Our code use the Virtual Brain https://www.thevirtualbrain.org/tvb/zwei/home (version 2.9)

For manuscripts utilizing custom algorithms or software that are central to the research but not yet described in published literature, software must be made available to editors and reviewers. We strongly encourage code deposition in a community repository (e.g. GitHub). See the Nature Portfolio guidelines for submitting code & software for further information.

Data

Policy information about availability of data

All manuscripts must include a data availability statement. This statement should provide the following information, where applicable:

- Accession codes, unique identifiers, or web links for publicly available datasets
- A description of any restrictions on data availability
- For clinical datasets or third party data, please ensure that the statement adheres to our policy

All the experimental data used in this article in openly available and have been previously published. For the structure-function correlation analysis for Propofol can be found in (Jang et al., 2024), for Ketamine (Huang et al., 2023) and Sleep in (Stikvoort et al., 2024). The structural connectivity matrix can be found in (Larivière et al.,2021). The connectivity data used for the whole brain simulations can be found in https://github.com/mariasacha/paper_pipeline_hub/tree/master/TVB/tvb_model_reference/data/QL_20120814. Source data for Figures 3, 4, 5, and 6 are available with this manuscript.

Human research participants

Policy information about studies involving human research participants and Sex and Gender in Research.

Reporting on sex and gender	No data from human were collected during this study
Population characteristics	No data from human were collected during this study
Recruitment	No data from human were collected during this study
Ethics oversight	No data from human were collected during this study, and no ethical overisght was needed

Note that full information on the approval of the study protocol must also be provided in the manuscript.

Field-specific reporting

Please select the one below that is the best fit for your research. If you are not sure, read the appropriate sections before making your selection.

☒ Life sciences ☐ Behavioural & social sciences ☐ Ecological, evolutionary & environmental sciences

For a reference copy of the document with all sections, see nature.com/documents/nr-reporting-summary-flat.pdf

Life sciences study design

All studies must disclose on these points even when the disclosure is negative.

Sample size	For the simulations that were compared with experimental data, different seeds were used to introduce variability in the results. For the case of structural and functional connectivity analysis, the number of seeds was selected in order to match the number of available human samples. For the case of PCI analysis, we chose a sufficiently large number of seeds that was permitted by the restrictions of time and computational resources
Data exclusions	No data were excluded
Replication	To ensure replication, all the scripts that were used for the simulations are accessible to the readers
Randomization	Not relevant to our study that involves simulations
Blinding	Not relevant to our study that involves simulations

Reporting for specific materials, systems and methods

We require information from authors about some types of materials, experimental systems and methods used in many studies. Here, indicate whether each material, system or method listed is relevant to your study. If you are not sure if a list item applies to your research, read the appropriate section before selecting a response.

Materials & experimental systems

n/a	Involved in the study
☒	☐ Antibodies
☒	☐ Eukaryotic cell lines
☒	☐ Palaeontology and archaeology
☒	☐ Animals and other organisms
☒	☐ Clinical data
☒	☐ Dual use research of concern

Methods

n/a	Involved in the study
☒	☐ ChIP-seq
☒	☐ Flow cytometry
☒	☐ MRI-based neuroimaging
